# Somatic mosaic* SOX10* indel mutations underlie a form of segmental schwannomatosis

**DOI:** 10.1007/s00401-023-02641-6

**Published:** 2023-10-11

**Authors:** Merryl Terry, Rohit Gupta, Ajay Ravindranathan, Jasper Wu, Emily Chan, Andrew W. Bollen, Susan M. Chang, Mitchel S. Berger, Line Jacques, David A. Solomon

**Affiliations:** 1 Department of Pathology, University of California, San Francisco, San Francisco, CA USA; 2 Department of Neurological Surgery, University of California, San Francisco, San Francisco, CA USA

While the majority of schwannoma nerve sheath tumors are solitary sporadic tumors, a subset arise as part of heritable tumor predisposition syndromes termed schwannomatosis [11]. Neurofibromatosis type 2 (NF2, also now termed *NF2*-related schwannomatosis) is an autosomal dominant syndrome caused by heterozygous germline mutation in the *NF2* gene on chromosome 22q12.2, which encodes the Merlin protein [16]. Patients with NF2 often develop bilateral vestibular schwannomas, as well as non-vestibular schwannomas, multiple meningiomas, and spinal ependymomas [4]. Two other forms of autosomal dominant schwannomatosis are caused by heterozygous germline mutations in either the *SMARCB1* gene on chromosome 22q11.23 (which encodes the chromatin remodeling factor INI1/BAF47) or the *LZTR1* gene on chromosome 22q11.21 (which encodes a substrate-specific adaptor of CUL3-dependent ubiquitin ligase that negatively regulates Ras signaling) [7, 10]. Patients with *SMARCB1*- and *LZTR1*-associated schwannomatosis often develop multiple painful non-vestibular schwannomas in the absence of meningiomas or other tumor types [8, 14]. Germline mutation/deletion of the *CDKN2A* gene on chromosome 9p21.3 (which encodes a negative regulator of the cell cycle p16^INK4a^) or the *DGCR8* gene on chromosome 22q11.21 (which encodes a subunit of the microRNA processing complex) causes rare tumor predisposition syndromes that may be associated with development of multiple schwannoma or schwannoma-like nerve sheath tumors [1, 12, 13]. However, many patients and families with schwannomatosis do not have identifiable germline variants in *NF2*, *SMARCB1*, *LZTR1*, *CDKN2A*, or *DGCR8,* and efforts have been underway to identify other responsible molecular drivers of schwannoma predisposition [9, 15, 18]. While some individuals develop multiple schwannomas diffusely throughout the peripheral nervous system due to a germline mutation in one of the known schwannomatosis genes, other individuals develop multiple schwannomas that are limited to a segment of the body [11]. Such “segmental schwannomatosis” is presumed to be caused by somatic mosaicism (also termed constitutional mosaicism or post-zygotic mosaicism) for a mutation acquired during embryogenesis or perhaps later during postnatal life [11]. The exact nature of such segmental schwannomatosis including the responsible molecular drivers and their timing of acquisition during human life are not well defined. Here, we report identification of somatic mosaicism for *SOX10* indel mutations as the genetic alteration underlying a form of segmental schwannomatosis.

A 41-year-old female initially presented with progressively worsening left leg and foot pain (Fig. 1a). Examination revealed fullness of the left thigh and an absent left ankle reflex. MR imaging showed several nodular masses along the course of the sciatic nerve in the mid-thigh (Fig. 1b). Following excision, numerous new nodules developed along the length of the left sciatic nerve with a “beads on a string” imaging appearance (Fig. 1b). She underwent four additional surgical excisions over the next 20 years due to continued pain and paresthesia. A second 49-year-old female initially presented with neck and shoulder pain. Imaging revealed two well-circumscribed and anatomically discrete masses in the left neck at levels 2 and 5 along the course of the left spinal accessory nerve (cranial nerve XI, Fig. 1c). She underwent surgical excision of both masses and has remained disease free at 4 years of follow-up. Neither patient had cutaneous neurofibromas, café-au-lait macules, or axillary and inguinal freckling. Neither patient had a family history of nerve sheath tumors. Neither patient had clinical features of Waardenburg syndrome type 4 associated with constitutional defects in the *SOX10* gene (Online Mendelian Inheritance in Man # 613266), including sensorineural hearing loss, abnormal pigmentation of the hair and skin, aganglionic megacolon (Hirschsprung disease), peripheral demyelinating neuropathy, central dysmyelinating leukodystrophy, and seizures/tremors. Histopathologic evaluation of the multiple resected tumors in both patients revealed schwannomas with classic histological features including both compact Antoni A and loose microcystic Antoni B areas, along with diffuse positivity for S100 and SOX10 immunostaining (Fig. 1d, e, Supplementary Figs. S1, S2). There was diffuse positivity for SMARCB1/BAF47/INI1 expression, without the pattern of mosaic loss that has been reported in some schwannomatosis-associated schwannomas (Supplementary Fig. S2) [3].

**Fig. 1:**
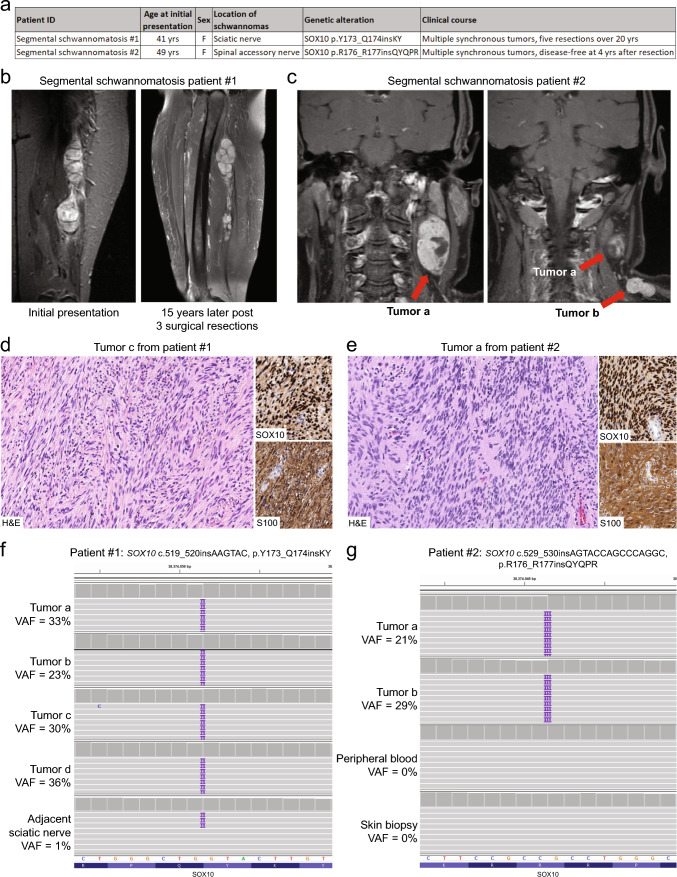
Segmental schwannomatosis arising from somatic mosaic *SOX10* indel mutations. **a** Clinicopathologic features of the two patients with segmental schwannomatosis arising due to somatic mosaic *SOX10* indel mutations. **b**, **c** Imaging of the two patients showing multiple synchronous schwannomas along the left sciatic nerve of patient #1 and the left spinal accessory nerve of patient #2 at time of initial presentation. **d**, **e** Histologic features of the schwannomas arising in the setting of somatic mosaic *SOX10* indel mutations. **f**, **g** Snapshots of the somatic mosaic *SOX10* indel mutations present in the two patients from genomic profiling performed on multiple independent tumor samples and paired normal samples for each patient. *VAF* variant allele frequency

Genomic analysis was performed on four tumor specimens and adjacent uninvolved sciatic nerve tissue as a source of non-neoplastic constitutional DNA for the first patient, and the two tumor specimens along with both peripheral blood and a skin biopsy specimen as a source of constitutional DNA for the second patient. The multiple tumors from both patients were found to harbor short in-frame insertion/duplication mutations in the *SOX10* gene (Supplementary Table S1), similar to those recently discovered in approximately 30% of sporadic solitary schwannomas that were localized at the carboxy-terminal end of the HMG-box DNA binding domain of the encoded homeobox transcription factor (Supplementary Fig. S3) [18]. The first patient harbored a p.Y173_Q174insKY (also annotated as p.K172_Y173dup) mutation that had been found in several sporadic schwannomas, while the second patient harbored a p.R176_R177insQYQPR mutation which was also previously identified in the sporadic schwannoma cohort [18]. The identical *SOX10* mutation was present in each of the four tumors from the first patient, and the identical *SOX10* mutation was present in both tumors from the second patient (Fig. 1f, g). These *SOX10* indel mutations were absent from the non-neoplastic constitutional DNA samples from these patients, thereby proving their somatic origin. No chromosomal copy number aberrations were present beyond monosomy/loss of 22q (Supplementary Table S2), and no other genetic alterations characteristic of nerve sheath tumors were identified involving *NF1*, *NF2*, *SMARCB1*, *LZTR1*, *ERBB2*, *TRAF7*, *CDKN2A*, *TP53*, *SUZ12*, *EED*, *PRKAR1A*, or *VGLL3* [6, 17]. Genome-wide DNA methylation profiling using the Infinium EPIC Beadchips revealed that these tumors all epigenetically classified as schwannomas (Supplementary Table S3). Furthermore, these tumors clustered together with *SOX10*-mutant sporadic schwannomas which we previously found are epigenetically distinct from *NF2*-mutant schwannomas (Supplementary Fig. S4, Supplementary Table S4) [18].

We surmise that the *SOX10* indel mutations likely occurred in these patients in a neural crest or Schwann cell progenitor during embryogenesis or early postnatal life. This resulted in individuals that harbor these *SOX10* mutations in Schwann cells and their progenitors in a limited segmental distribution along a single peripheral nerve, which then gave rise to multiple genetically identical schwannomas over time. We speculate that the absence of Waardenburg syndrome type 4 clinical features in these individuals is because the somatic mosaicism for the *SOX10* indel mutations was limited to a small population of neural crest progenitor cells affecting only a single peripheral nerve and not the central or autonomic nervous systems. The *SOX10* gene encodes a homeobox transcription factor known to be critical for differentiation of Schwann cells and maturation to a myelinating cell state [2, 5]. Our prior studies in a fetal glial cell model found that *SOX10* indel mutations impair transactivation of glial differentiation and myelination genes, and likely cause schwannoma development through blockade of Schwann cell maturation [18]. Based on the observations in these two patients, we conclude somatic mosaicism for *SOX10* indel mutations causes a form of segmental schwannomatosis lacking other known nerve sheath tumor molecular alterations.

### Supplementary Information

Below is the link to the electronic supplementary material.

Supplementary file1 (PDF 7465 KB)

Supplementary file2 (XLSX 19 KB)

## Data Availability

Raw and processed DNA methylation data from the segmental schwannomatosis tumor cohort has been deposited at the NCBI Gene Expression Omnibus (GEO) under accession number GSE239715. Digitally scanned image files of representative H&E and immunostained sections from the schwannomas are available at the following link: https://figshare.com/projects/Segmental_schwannomatosis/175770. Annotated DNA sequencing data from the schwannoma cohort are provided in the supplementary data tables. Raw sequencing data files are available from the authors upon request.
