# Supplementary material for: Somatic mosaic SOX10 indel mutations underlie a form of segmental schwannomatosis
Source: Acta Neuropathol. 2023 Oct 11;146(6):857–60. doi: 10.1007/s00401-023-02641-6 (PMC10627975; doi:10.1007/s00401-023-02641-6)
Supplement: Supplementary file 1 — Supplementary file1 (PDF 7465 KB) [file 401_2023_2641_MOESM1_ESM.pdf]

Segmental schwannomatosis patient #1: 41 year old female, numerous schwannomas along left sciatic nerve over 20 years, somatic mosaic *SOX10* p.Y173_Q174insKY mutation

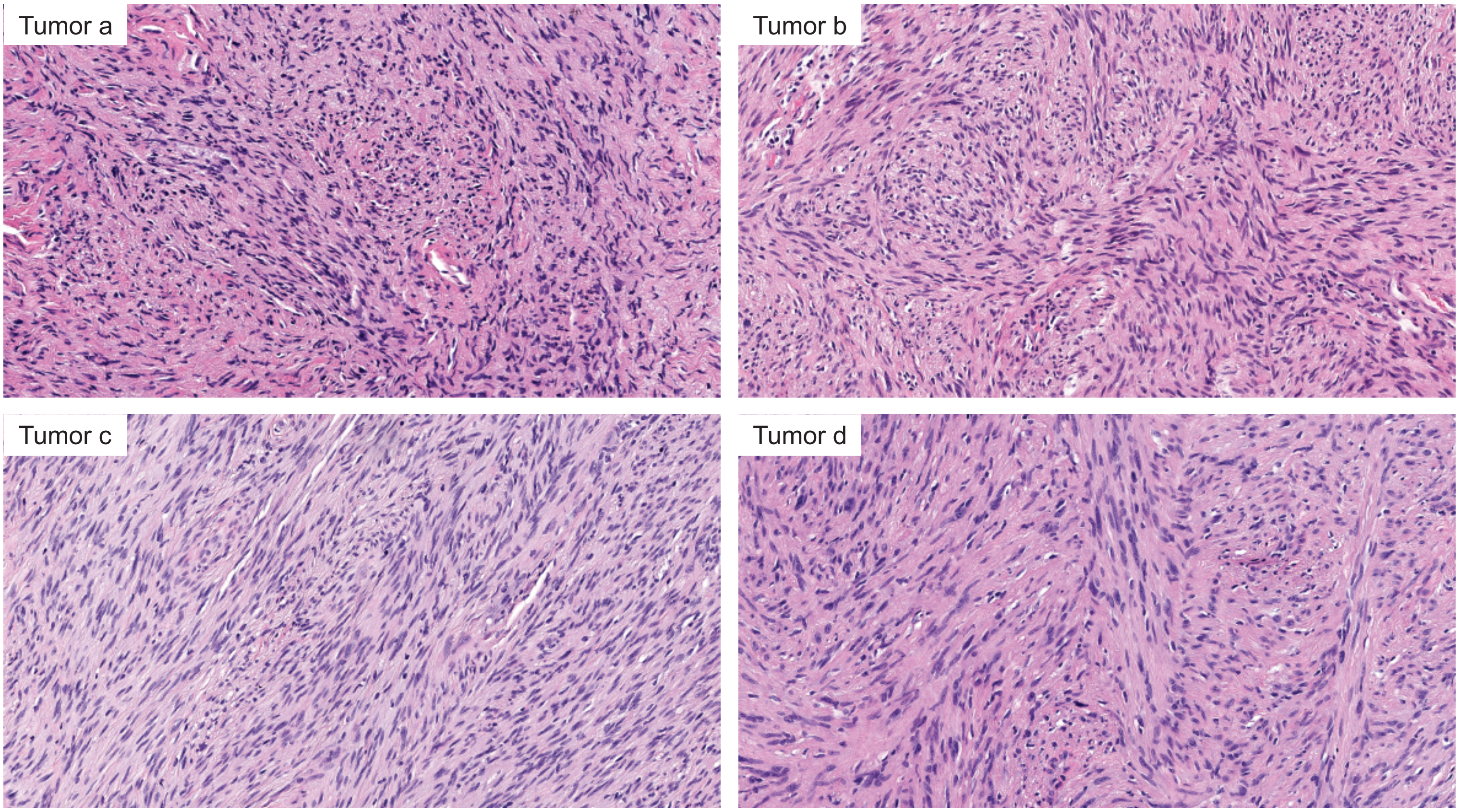

Segmental schwannomatosis patient #2: 49 year old female, two synchronous schwannomas along left spinal accessory nerve, somatic mosaic *SOX10* p.R176_R177insQYQPR mutation

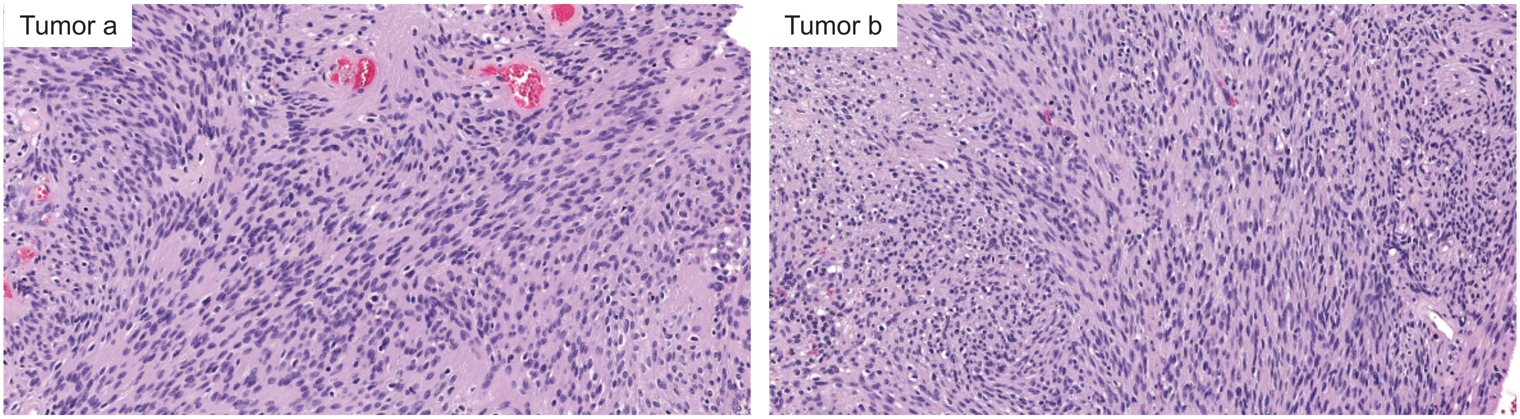

Supplementary Figure 1. Representative H&E-stained sections of schwannomas arising in the setting of segmental schwannomatosis due to somatic mosaic *SOX10* indel mutations.

Segmental schwannomatosis patient #1, tumor d

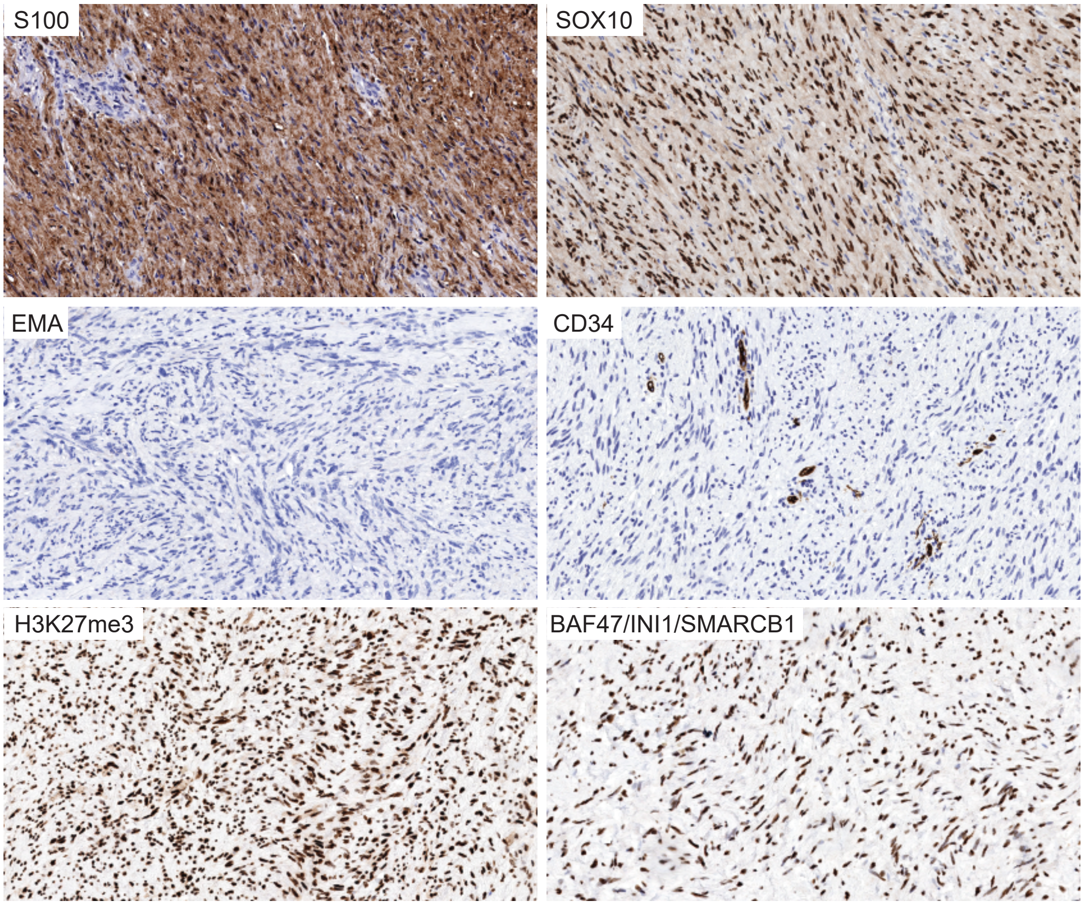

Segmental schwannomatosis patient #2, tumor a

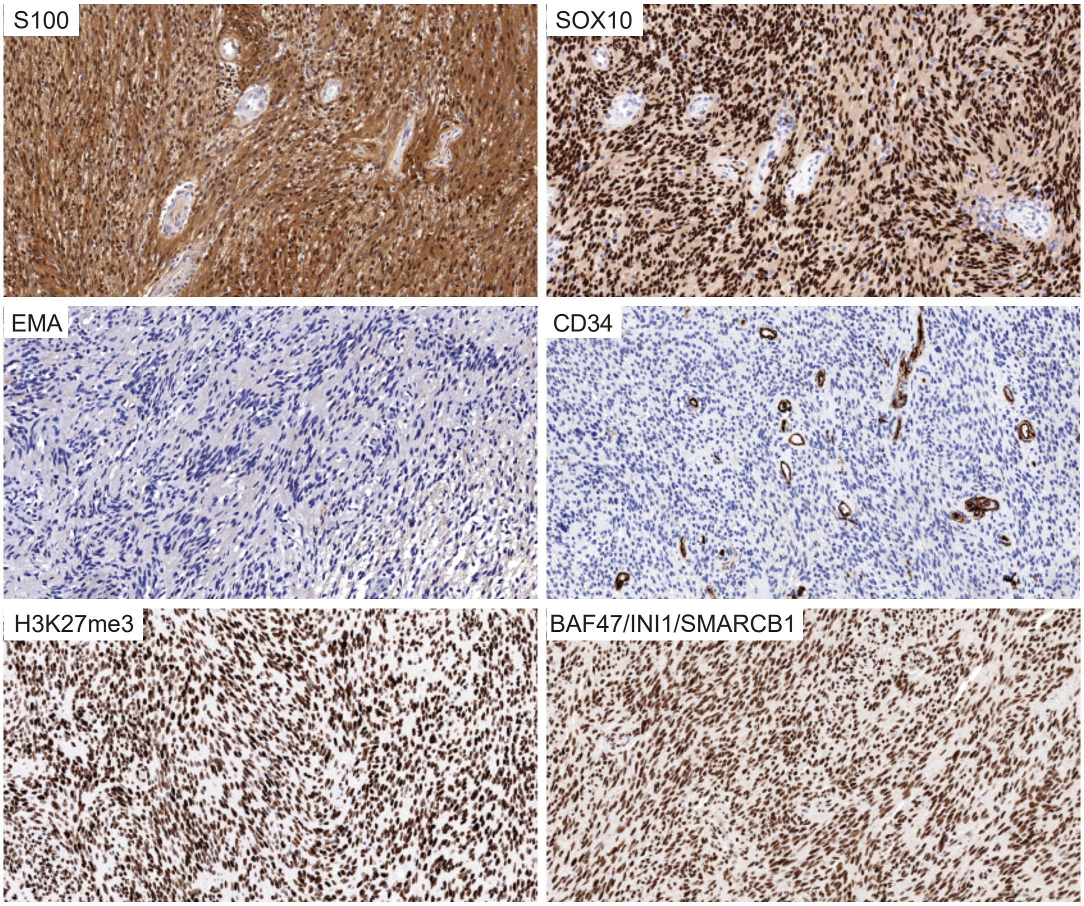

Supplementary Figure 2. Immunohistochemical features of schwannomas arising in the setting of segmental schwannomatosis due to somatic mosaic *SOX10* indel mutations.

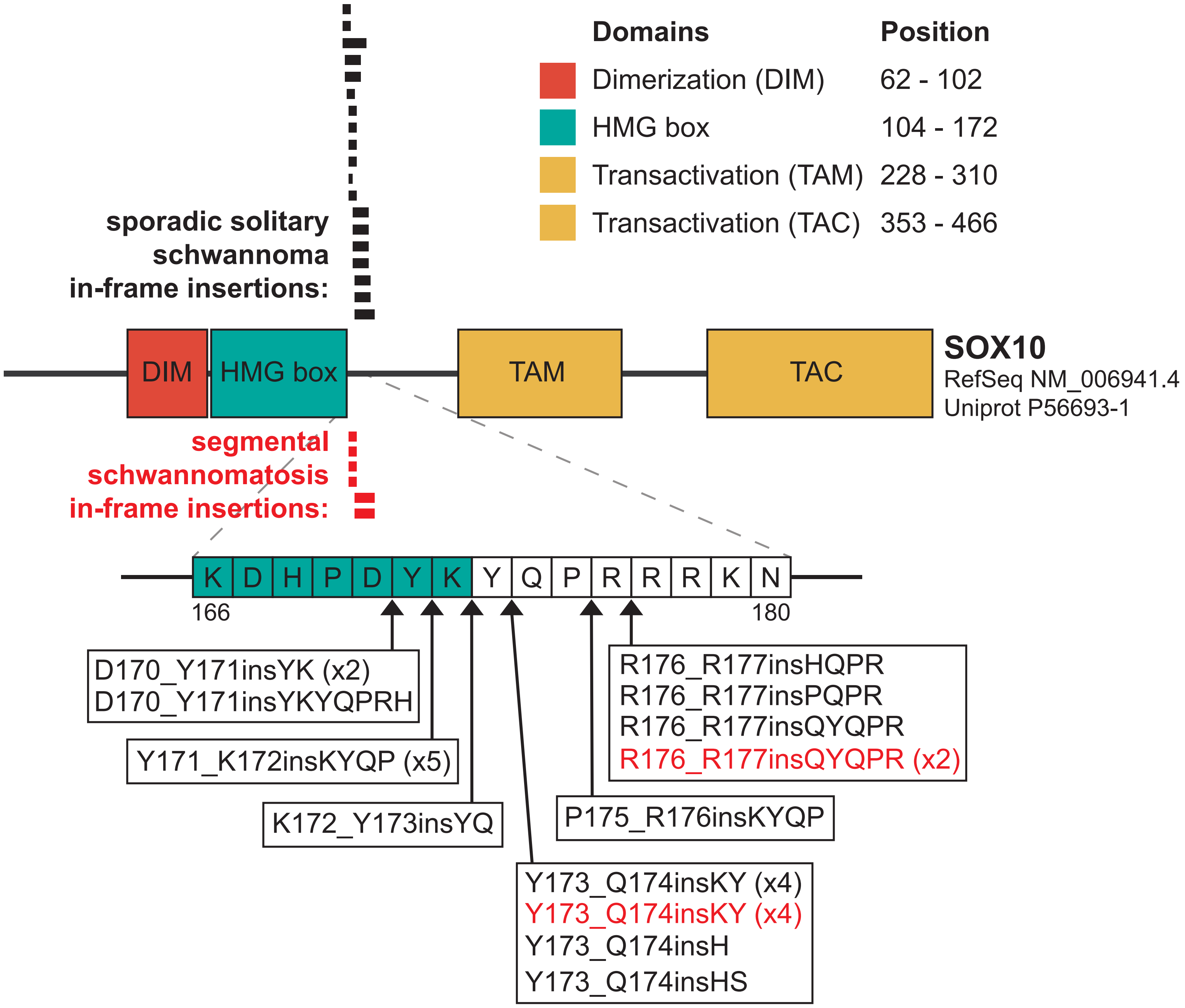

Supplementary Figure 3. Diagram of the SOX10 protein illustrating the recurrent in-frame insertion/duplication mutations in both sporadic solitary schwannomas (colored black) and segmental schwannomatosis-associated schwannomas (colored red). These in-frame indel mutations all localize at the carboxy-terminal end of the HMG box DNA binding domain (shaded green) of the SOX10 homeobox transcription factor.

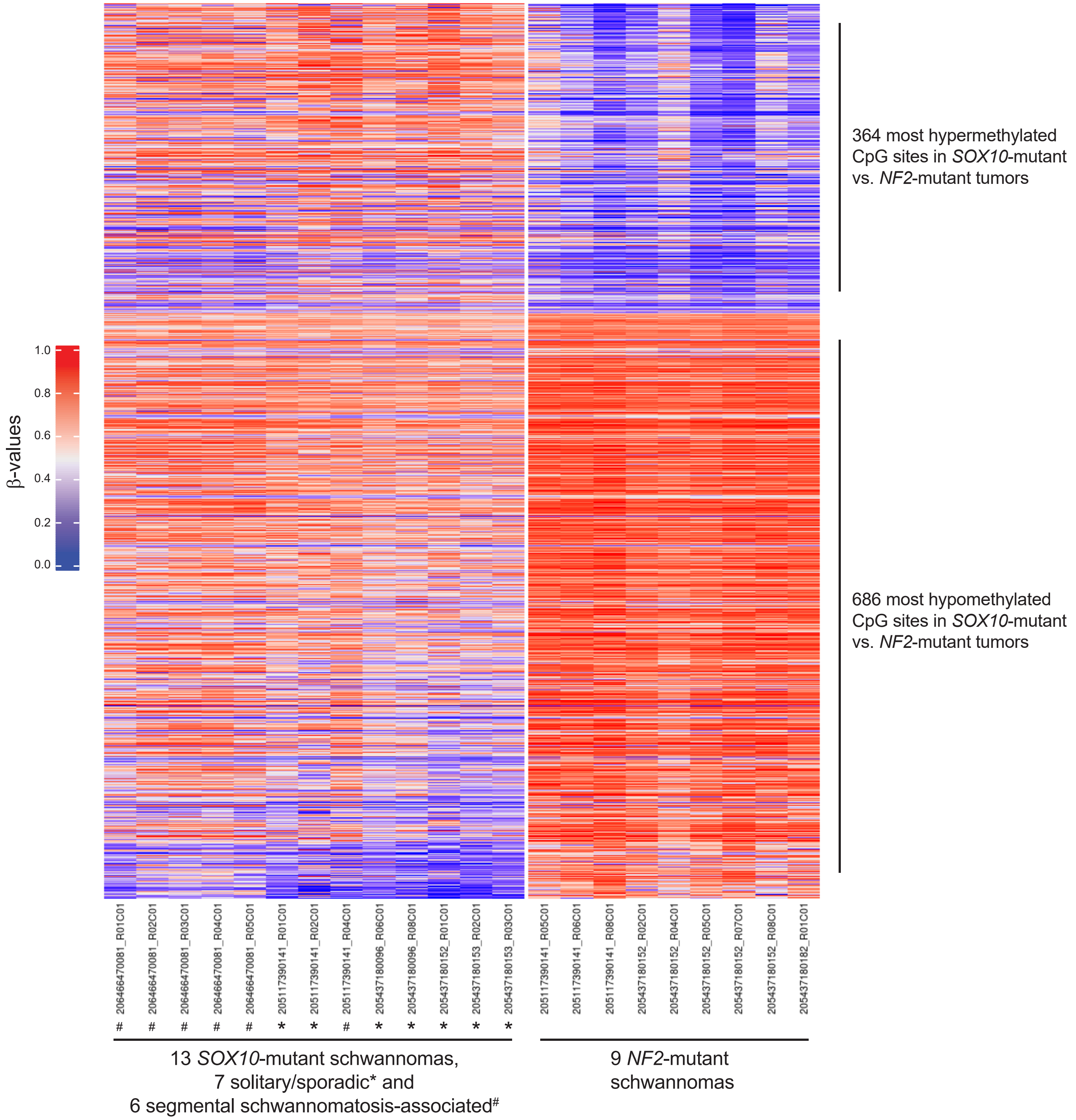

Supplementary Figure 4. Segmental schwannomatosis-associated schwannomas arising in the setting of somatic mosaic *SOX10* indel mutation share a similar epigenetic signature with solitary sporadic schwannomas with *SOX10* indel mutation, and are epigenetically distinct compared to those schwannomas with *NF2* mutation. Shown is unsupervised hierarchical clustering of Infinium EPIC DNA methylation data for 6 segmental schwannomatosis-associated schwannomas with somatic mosaic *SOX10* indel mutations, 7 solitary sporadic schwannomas with *SOX10* indel mutations, and 9 schwannomas with inactivating *NF2* mutations. Hierarchical clustering segregated these tumors into 2 epigenetic groups, which comprised those with *SOX10* indel mutation and those with *NF2* mutation. Shown is a heatmap of the 1,050 most differentially methylated probes amongst the 22 tumors. See Supplementary Table S4 for sample manifest.
